# Targeting epigenetics for cancer therapy

**DOI:** 10.1007/s12272-019-01126-z

**Published:** 2019-02-26

**Authors:** Jong Woo Park, Jeung-Whan Han

**Affiliations:** Research Center for Epigenome Regulation, School of Pharmacy, Sungkyunkwan University, Suwon, 16419 Republic of Korea

**Keywords:** Cancer epigenetics, DNA methylation, Histone modification, Epigenetic drugs

## Abstract

Cancer can be identified as a chaotic cell state, which breaks the rules that govern growth and reproduction, with main characteristics such as uncontrolled division, invading other tissues, usurping resources, and eventually killing its host. It was once believed that cancer is caused by a progressive series of genetic aberrations, and certain mutations of genes, including oncogenes and tumor suppressor genes, have been identified as the cause of cancer. However, piling evidence suggests that epigenetic modifications working in concert with genetic mechanisms to regulate transcriptional activity are dysregulated in many diseases, including cancer. Cancer epigenetics explain a wide range of heritable changes in gene expression, which do not come from any alteration in DNA sequences. Aberrant DNA methylation, histone modifications, and expression of long non-coding RNAs (lncRNAs) are key epigenetic mechanisms associated with tumor initiation, cancer progression, and metastasis. Within the past decade, cancer epigenetics have enabled us to develop novel biomarkers and therapeutic target for many types of cancers. In this review, we will summarize the major epigenetic changes involved in cancer biology along with clinical and preclinical results developed as novel cancer therapeutics.

## Introduction

According to the Centers for Disease Control and Prevention (CDC), cancer is the second leading cause of death in the top ten diseases, next to heart disease (Heron et al. 2012). Although we have accumulated vast knowledge about cancer, the statistics show that we are still far from overcoming cancer. What makes cancer so hard to overcome and how much do we know about cancer? Until early 2000s, cancer was considered as a set of diseases caused by the accumulation of genetic mutations that control normal cellular homeostasis (Vogelstein et al. 2013). Oncogenes and tumor suppressor genes (TSGs) are the most well-known classes of genes implicated in cancer (Zhu et al. 2015). Proto-oncogenes, which normally help to regulate cell growth or differentiation, can become oncogenic by genetic mutation. Point mutation, chromosomal mutation, or copy number variation can lead to oncogene activation through amplified expression or gain-of-function from protein structural rearrangement. Translocation of the Philadelphia (Ph) chromosome in chronic myeloid leukemia (CML) was discovered in 1960 (Nowell 2007). Translocation of proto-oncogene *ABL* at 9q34 to *BCR* on chromosome 22 can produce a fusion gene called *BCR*-*ABL1*, coding for a hybrid oncoprotein (Rowley 2001; Imbach 2014). BCR-ABL1 fusion oncoprotein is a constitutively active tyrosine kinase signaling protein, causing the cell to divide uncontrollably and therefore develop CML. Ras mutation is another most well-known gain-of-function mutation identified in human cancer (Bos 1989; Fernández-Medarde and Santos 2011; Prior et al. 2012). RAS proteins (KRAS, NRAS, and HRAS) function as GDP–GTP-regulated binary on–off switches, which regulate cytoplasmic signaling networks that are responsible for proliferation and cell survival (Bos 1989). Mutation of RAS proteins at 12, 13, or 61 codon enhances the binding of GTP to the Ras protein, resulting in constitutive activation of Ras, which is associated with hyperproliferative developmental disorders and cancer. Among three isoforms, K-Ras has been shown to be the most frequently mutated isoform in most cancers. K-Ras gene is found to be mutated in 22% of all tumors, especially 90% of pancreatic tumors (Forbes et al. 2011).

In contrast to oncogenes that are activated mainly by gain-of-function mutations, tumor suppressors lose their functions (loss-of-function mutation) through deletions or point mutations. The retinoblastoma protein (RB) is a tumor suppressor protein, which mutation was originally identified in a rare childhood cancer retinoblastoma (Knudson 1971). Rb can suppress cellular proliferation by regulating the E2F transcription factor, and the Rb/E2F pathway plays a critical role in the initiation of DNA replication (Nevins 2001). Later studies have identified complex molecular functions of Rb through interactions with various proteins, and the Rb/E2F pathway was found to be functionally inactivated in virtually all human cancers (Chinnam and Goodrich 2011; Dyson 2016).

The functions of proto-oncogene proteins are to enhance cell division or inhibit cell death, while the functions of tumor suppressors are normally to prevent cell division or cause cell death. Therefore, either gain-of-function mutations of proto-oncogenes or loss-of-function mutations of tumor suppressors could initiate cancer through uncontrolled cell growth and defective apoptosis (Zhu et al. 2015). After the Human Genome Project (HGP) was completed, we achieved a great deal in human genetics, and it became the starting point for human genomics (Gonzaga 2012; Hood and Rowen 2013). Moreover, large-scale cancer genome projects, such as The Cancer Genome Atlas (TCGA), the Wellcome Trust Sanger Institute’s Cancer Genome Project, and the International Cancer Genome Consortium (ICGC), have shed light on cancer genomics. In addition, somatic mutations from thousands of tumors have provided insights into cancer development processes along with available therapeutic targets for cancer (McLendon et al. 2008; Hudson et al. 2010; Pleasance et al. 2010).

Achievements of HGP and other big studies have been powerful; however, the sequence itself does not explain how the genome is packaged into chromatin and provide differential expression of genes for proliferation, development, and differentiation. Therefore, the current paradigm to explain cancer development has now expanded to cancer genetics and epigenetics. While cancer genetics focus on abnormal gene expression, including altered protein expression by either deletion or amplification mutations, cancer epigenetics focus on the regulation of gene expression without changing the genome sequence. Altered gene expression in cancer through epigenetic pathways is very complex and is determined by chromatin structure changes, including DNA methylation, histone variants and various modifications, nucleosome remodeling, and small non-coding RNAs (Dawson and Kouzarides 2012). This review highlights the basic principles of epigenetic pathways involved in cancer development along with recent progress in clinical and preclinical studies targeting cancer epigenetics.

## DNA methylation

Epigenetic control is the way to determine which genes should be turned on or off for normal development and in response to the environment. They are mostly regulated by groups of proteins called ‘epigenetic writers’, ‘epigenetic readers’, and ‘epigenetic erasers.’ The writer is the enzyme that creates modifications around the genome. This change is recognized by the reader. Finally, when the epigenetic change is no longer needed, erasers can remove it.

DNA methylation was the first epigenetic modification found in humans in the early 1980s (Cooper 1983; Doerfler 1983). DNA methylation occurs in cytosines of CpG (Cytosine-phosphate-Guanine) dinucleotide sequences to create 5-methylcytosine (5mC), which is catalyzed by DNA methyltransferases (DNMTs) using *S*-adenyl methionine (SAM) as the methyl donor. Promoter regions containing higher GC content are called CpG islands (CGIs). Hypermethylation of CGIs occurs in heterochromatin regions, while hypomethylation commonly occurs in actively expressed genes (Ohm et al. 2007; Meissner et al. 2008). DNA methylation of CGIs can be found at many different locations within the genome, including centromeres, telomeres, and inactive X-chromosomes (Vera et al. 2008; Pasque et al. 2018; Skakkebæk et al. 2018). There are three identified DNMT enzymes, which are DNMT1, DNMT3A, and DNMT3B. DNMT3A and DNMT3B are de novo methyltransferases that are responsible for the initial CpG methylation during embryogenesis (Okano et al. 1998). DNMT1 maintains the methylation pattern during chromosome replication by preferential methylation on hemimethylated CpGs. After CpG methylation, 5mC can become a platform for several methyl-CpG-binding domain (MBD) proteins, such as MBD1, MBD2, MBD3, MBD4, and MeCP2, for further chromatin-templated processes (Mashimo et al. 2013). There are other MBD-containing proteins, such as MBD5/6, SETDB1/2, and BAZ2A/B. The MBD proteins cooperate with other epigenetic proteins like histone modifying enzymes or chromatin remodeling complexes at the 5mC region and facilitate transcriptional repression (Du et al. 2015a).

Although direct removal of DNA methylation has not been detected so far, there are a few ways to remove DNA methylation. First, passive DNA demethylation through steady dilution of methylation patterns can happen by replication (Kriukiene et al. 2012). Secondly, ten–eleven translocation (TET 1–3) enzymes can oxidize 5mCs to create 5-hydroxymethylcytosine (5hmC), and subsequently formyl-(5-fc) and carboxyl-(5caC) derivatives are formed. The derivatives finally can be excised by the DNA repair protein thymine glycosylase (TDG) to be replaced by unmodified cytosine via the base excision repair (BER) pathway (Kohli and Zhang 2013).

Aberrant DNA methylation patterns, both hyper- and hypo-methylation, have been reported in many different types of cancer, including prostate, breast, gastric, liver, lung, glioblastoma, and leukemia (Sun et al. 2010; Barbano et al. 2013; Chao et al. 2013; Mehta et al. 2015; Liu and Brenner 2016; Cecotka and Polanska 2018; Klughammer et al. 2018). First cancer implication was the global hypomethylation at CpG sites of DNA repetitive elements identified in tumor cells (Bedford and van Helden 1987; Lin et al. 2001). Loss of imprinting at the insulin-like growth factor 2 (*IGF2*) gene locus is frequently observed in cancer and is provided as a colon cancer diagnosis (Cui et al. 2002). Conversely, hypermethylation of specific genes have also been identified to explain the role of DNMTs in tumorigenesis. Hypermethylation of CpG islands in TSG promoters, including *Braca1*, *Rb,* or *p53* promoters, leads to inactivation of each protein and can enhance cancer development (Rideout et al. 1991; Sakai et al. 1991; Baldwin et al. 2000). Alteration of normal DNA methylation has been well profiled for over 25 years of epigenetic studies and provides its application for diagnostic and therapeutic targets (Heyn and Esteller 2012). Although the exact cause of deregulated DNA methylation patterns in cancer is not yet well established, an accumulation of data has shown that either mutation or overexpression of DNMT proteins and MBD protein is correlated with tumorigenesis (Du et al. 2015b; Spencer et al. 2017). In addition, several reports have emerged that mutations of TET family genes were found in numerous hematological malignancies (Cimmino et al. 2011; Nakajima and Kunimoto 2014).

Targeting of aberrant DNA methylation patterns has been attempted, and two cytidine analogs, 5-azacytidine/vidaza (AZA) and 5-aza-2′-deoxycytidine/dacogen (DAC), have been approved for the treatment of myelodysplastic syndromes (MDS) by the FDA (Raj and Mufti 2006; Santos et al. 2010). These two compounds form an irreversible covalent complex with DNMT1 and trigger proteasome-mediated DNMT1 degradation. Second-generation analog guadecitabine (SGI-110), which is an active metabolite of decitabine, is being tested in clinical trial for MDS and acute myeloid leukemia (AML) (Kantarjian et al. 2017). Although the role of the TET family in several cancers has been suggested from recent studies, a TET protein inhibitor has yet to be tested for cancer treatment.

Writers, readers, and eraser enzymes for DNA methylation and inhibitors are summarized in Table 1.

**Table 1:** Epigenetic drugs against DNA methylation changes

	Writer (DNMTs)	Reader (MBD family)	Eraser (TET family)
Enzyme	**DNMT1**, DNMT3a, DNMT3b	MBD1, MBD2, MBD3, MBD4, MeCP2, MBD5/6, SETDB1/2, BAZ2A/B	TET1, TET2, TET3
Drugs	5-azacytidine (approved) 5-aza-2′-deoxycytidine (approved) SGI-110 (clinical trials)		

Enzymes for the drug target are highlighted in bold

## Histone modification-lysine acetylation

DNA within eukaryotic cells is packaged as chromatin, and the histone octamer is the central component of the nucleosomal subunit. The histone subunit in the nucleosome possesses a characteristic tail, which contains specific amino acid residues for covalent posttranslational modifications (PTMs), such as acetylation, methylation, phosphorylation, ubiquitylation, sumoylation, or ADP ribosylation. Each epigenetic PTM cooperates to regulate chromatin states.

Histone acetylation is crucial for active gene transcription to influence the compaction state of chromatin by neutralizing basic charges on unmodified lysine residues, decreasing the electrostatic interaction between negatively charged DNA and histones. Histone acetylation occurs on the lysine residue, balanced by two enzymes: histone acetyltransferase (HAT) and histone deacetylases (HDAC). There are primarily three families of HAT enzymes, including GNAT family (Gcn5, PCAF, Hat1), MYST family (MOZ/Morf, Ybf2, Sas2, Tip60), and CBP/P300 family (p300/CBP, Taf1) (Marmorstein and Roth 2001). These enzymes are also known to acetylate hundreds of other proteins besides histones, such as p53, sTAT3, GATA, etc., and have numerous biological functions, including regulation of protein stability, DNA binding affinity, and protein interactions (Spange et al. 2009). As epigenetic erasers, 18 HDAC isoforms have been identified in humans. Class I (HDACs 1, 2, 3, 8), Class IIa (HDACs 4, 5, 7, 9), Class IIb (HDACs 6, 10), and Class IV (HDAC11) are classical HDAC families that require a zinc ion (Zn^2+^) for their actions, whereas Class III HDACs (SIRT1 to 7) require NAD^+^ and are Zn^2+^-independent (Zhang et al. 2015). Aberrant histone lysine acetylation patterns, especially loss of histone H4 lysine (K) 16 acetylation, have been reported as a common hallmark of human cancer (Fraga et al. 2005). There are numerous reports showing involvement of HAT mutation or loss-of-function with many diseases, including cancer. Truncation mutations and in-frame insertion mutations of EP300 have been identified in several different cancers (Gayther et al. 2000). Further, it has been reported that the genes for p300, CBP, MOZ, and MORF are rearranged in recurrent leukemia-associated chromosomal abnormalities (Yang 2004). Although involvement of dysregulated HAT in many diseases is becoming clear, clinical application of the HAT inhibitor was not successful.

In addition to histone deacetylation, HDACs have other roles in association with several transcription factors, tumor suppressors, and oncogenes. For example, HDAC1 forms a complex with Rb and E2F transcription factors and regulates gene expression of the cell cycle (Brehm et al. 1998; Kennedy et al. 2001). Moreover, increased expression of HDAC family proteins has been observed in many cancers, including B cell acute lymphoblastic leukemia (ALL) and T cell ALL, indicating the role of histone acetylation in various leukemogenesis (Moreno et al. 2010; Tao et al. 2013). Although HAT inhibitors were not clinically successful, HDACs have become great targets for anticancer agents. Five classes of compounds—(I) hydroxamic acids; (II) short chain fatty acids; (III) benzamides; (IV) cyclic tetrapeptides; and (V) sirtuin inhibitors—are currently developed as anticancer reagent, and they are either isoform-selective or pan-inhibitors. Among hydroxamates, SAHA, Belinostat and Panobinostat are approved for T cell lymphoma. Romidespsin is a cyclicpeptide HDAC inhibitor which is approved for cutaneous T cell lymphoma (CTCL) and peripheral T-cell lymphoma (PTCL). The short chain fatty acid, Valproic acid, is approved for epilepsy. Many other classes of HDAC inhibitors are in different clinical stages for various cancers (Eckschlager et al. 2017).

Acetylated histone can serve as a binding site for regulatory factors, chromatin-remodeling complexes, and especially for bromodomain-containing proteins, which are known as histone acetylation readers. The human genome encodes 61 bromodomains present in 46 proteins, which are HATs, histone methyltransferases (HMTs), and transcription initiation factors. Among these BRD proteins, Bromodomain and extra-terminal (BET) proteins (BRD2, BRD3, BRD4, BRDT) are highly associated with several types of cancer (Padmanabhan et al. 2016). Small molecule triazolodiazepine-based inhibitors of the BET bromodomain, JQ1 and I-BET, were first developed (Pérez-Salvia and Esteller 2017). They selectively bind to bromodomains BD1 and BD2 of the BET family. BET inhibitors have shown great efficacy against human and murine MLL-fusion leukemic cell lines and mouse leukemia models. From further mechanistic studies, BET inhibition has been shown to suppress cancer through inhibition of Myc expression, targeting JAK-STAT, NF-κB pathway, and p53 acetylation (Chan et al. 2015; Huang et al. 2017; Xu and Vakoc 2017). Currently, many different BET inhibitors are in clinical trial phase I or II for various different types of cancers. Enzymes for histone acetylation, deacetylation, and readers are summarized in Table 2.

**Table 2:** Epigenetic drugs against histone acetylation changes

	Writer (HATs)	Reader (BRD family)	Eraser (HDACs)
Enzyme	GNAT family (Gcn5, PCAF, Hat1) MYST family (MOZ/Morf, Ybf2, Sas2, Tip60) CBP/P300 family (p300/CBP, Taf1)	**BET proteins (BRD2, BRD3, BRD4, BRDT)**	**Class I (HDACs 1, 2, 3, 8)** **Class IIa (HDACs 4, 5, 7, 9)** **Class IIb (HDACs 6, 10)** **Class IV (HDAC11)** Class III HDACs (SIRT1 to 7)
Drugs		JQ1 (preclinical), I-BET762(Clinical trials)	Belinostat (approved) SAHA (approved) Romidepsin (approved) Valproic acid (approved)

Enzymes for the drug target are highlighted in bold

## Histone modification-lysine and arginine methylation

Another well-known histone modification is histone methylation on arginine and lysine residues. Different from histone acetylation, methylation does not change the physical interaction between DNA and histone by neutralizing the histone charge. Further, methylation of specific lysine or arginine residues refers to either an active or repressive gene expression. Lysine methylation can exist in a mono-, di-, or tri-methylated state, implying the complexity of the regulatory mechanisms. Generally, H3 lysine 4 (H3K4), H3K36, and H3K79 methylation is correlated with active gene expression, while di- and tri-methylation of H3K9, H3K27, and H3K20 are linked to gene repression (Vermeulen et al. 2010).

Similar to other epigenetic modifications, histone methylation is also regulated by writer (lysine methyltransferases: KMTs), reader, and eraser (lysine demethylases: KDMs) proteins. KMTs are comprised of 51 SET (Su (var)3-9, Enhancer of Zeste, Trithorax) domain KMTs and one non-SET domain lysine HMT, known as DOT1L (Qian and Zhou 2006). DOT1L contains a catalytic domain, which is structurally related to the domains of protein arginine methyltransferases (Nguyen and Zhang 2011). SET-domain proteins transfer a methyl group from *S*-adenosyl-l-methionine (SAM) to the amino group of a lysine residue on the histone or other protein, leaving a methylated lysine residue and the cofactor byproduct S-adenosyl-l-homocysteine (SAH). Most KMTs can methylate several non-histone proteins, including p53, PCNA, STAT3, RARα, E2F1, FOXO3, DNMT1, and KMT1c (Moore and Gozani 2014). KDMs are comprised of two families of proteins based on the organization of their catalytic domains and the type of oxidative mechanisms for the demethylation reaction. The first group is the Jumonji (Jmjc) domain-containing KDM family, which utilizes 2-oxoglutarate (2-OG; α-ketoglutarate) as a co-factor. The second group is KDM1A (LSD1, BHC110, AOF2) and KDM1B (LSD2), which utilizes flavin adenine dinucleotide (FAD) as a co-factor for demethylation activity.

Aberrant histone lysine methylation patterns have been identified in various human cancers. For example, low levels of H3K4me2 correlated with low survival rates in both lung and kidney cancers and was also associated with adverse prognosis in non-small cell lung carcinomas (NSCLC), hepatocellular carcinomas (HCC), and breast cancers (Barlési et al. 2007; Elsheikh et al. 2009; Seligson et al. 2009). Either up- or down-regulated KMTs frequently found in cancer and KDMs are involved in tumorigenesis by several other mechanisms, including alteration of histone or non-histone protein methylation (Varier and Timmers 2011; Colón-Bolea and Crespo 2014). Dysregulation of H3K27me3 is frequently observed in many types of cancers, and overexpression of EZh2 or mutations in the SET domain of EZH2 have been reported in lymphomas, causing an increase of H3K27me3 (Pawlyn et al. 2017; Nienstedt et al. 2018). The histone demethylase LSD1 (KDM1A) is highly expressed in several cancers and is specifically required for terminal differentiation of hematopoietic cells (Sprüssel et al. 2012). By histone H3K4 1/2 demethylase activity, LSD1 (KDM1A) represses gene expression, but LSD1 can stimulate transcription through interaction with the androgen receptor (Metzger et al. 2005). Several LSD1 inhibitors, such as ORY-1001 or GSK2879552, have been developed and are under clinical trial for AML treatment (Maes et al. 2015).

Similar to any other PTMs, histone lysine methylation can serve as a recognition site for the ‘reader’ or effector proteins. The malignant brain tumor (MBT) domain protein, PHD (plant homeodomain) proteins, chromodomain proteins, PWWP domain, and WD40 repeat proteins are identified as histone lysine methylation readers (Herold et al. 2011). The inhibitor of growth (ING) family of tumor suppressor genes (ING1-5) contains a C-terminal PHD, which is known to preferentially bind di- and tri-methylated H3K4 and mediate many cellular processes (Champagne and Kutateladze 2009). Heterochromatin protein 1 (HP1) is another example of a methyl-lysine reader. Three isoforms of HP1 can interact with methylated H3K9 via its chromodomain. Much evidence has shown that not only alteration of histone modifying enzyme levels, but also alteration of methyl-lysine reader expression has cancer implications. For example, downregulation of HP1α has been linked to the higher invasive potential of breast cancer cells and papillary thyroid carcinoma (Wasenius et al. 2003; Norwood et al. 2006; De Koning et al. 2009).

Alterations in histone lysine methylation are tightly linked to the development of cancer and are suggested as potential cancer therapeutic targets. Many KMT inhibitors, such as DOT1L, EZH2, and SUV 39H1 inhibitors, are in preclinical or clinical trials. Many groups have developed EZH2 inhibitors, and among them, EPZ-6438 is in phase I/II trial for refractory B-cell lymphoma (Knutson et al. 2014). In addition, the DOT1L inhibitor EPZ-5676 is in phase I clinical trial for refractory AML and ALL (Lillico et al. 2018; Stein et al. 2018). However, the search for KMT or KDM inhibitors is still in its very first stages.

Histone arginine methylation is similar to lysine methylation in many ways. Protein arginine methyltransferases (PRMTs) also utilize SAM to transfer a methyl group to the guanidine nitrogen atoms of arginine to form methylarginines and SAH. There are three different forms of methylarginines: ω-N^G^-monomethylarginine (MMA), ω-N^G^,N^G^-asymmetric dimethylarginine (aDMA), and u-N^G^,N′^G^-symmetric dimethylarginine (sDMA). PRMTs can be subcategorized into three groups by their catalytic activity; type I (PRMT1, PRMT2, PRMT3, PRMT4, PRMT6, and PRMT8) and type II (PRMT5 and PRMT9) enzymes initially forms MMA as an intermediate before the establishment of aDMA or sDMA, respectively, while type III (PRMT7) enzymes only catalyze to form MMA (Yang and Bedford 2013). Generally, H4R3me2a, H3R2me2s, H3R17me2a, and H3R26me2a are correlated with active gene expression, while H3R2me2a, H3R8me2a, H3R8me2s, and H4R3me2s are linked to gene repression. PRMTs also can methylate many non-histone proteins that have arginine- and glycine-rich (GAR) motifs. RNA-binding proteins (RBPs), Tumor suppressor 53-binding protein 1 (53BP1), and many other proteins have been identified as substrates for PRMTs, and arginine methylation of these proteins is involved in various biological processes, such as transcription, cell signaling, mRNA translation, DNA damage signaling, receptor trafficking, protein stability, and pre-mRNA splicing (Wei et al. 2014).

Arginine methylation is a very stable modification; therefore, the existence of direct arginine demethylases is still controversial. Jumonji domain-containing protein, JmjD6, was reported to demethylate H3R2me2 and H4R3me2, but later it was identified as a lysine hydroxylase (Webby et al. 2009). Moreover, a recent study showed that one of peptidylarginine deiminases (PAIDs) protein PADI4 was recruited to the pS2 promoter region just prior to H3R17me2a loss, suggesting that it is responsible for removing this methyl mark (Denis et al. 2009). However, PADIs catalyze the deimination of arginine; therefore, they are not considered as “true” demethylases.

As epigenetic readers of arginine methylation, Tudor domain-containing proteins, such as SMN (Survival of motor neuron), SPF30 (Splicing factor 30), and TDRD1/2/3/6/9/11, have been identified to interact with methyl-arginine residues (Gayatri and Bedford 2014). However, the biological role of the interaction between these two is still unclear.

Aberrant expression of PRMT or dysregulation of PRMT activity are associated with several diseases, including many types of cancers. For example, PRMT1 is the major PRMT, which is responsible for 90% of arginine methylation, and it is upregulated in breast cancer, bladder cancer, pediatric ALL, etc. (Yoshimatsu et al. 2011; Zou et al. 2012). Most other PRMTs are also found to be upregulated in various types of cancers; as a result, PRMTs are attractive cancer targets. Recently, a few PRMT inhibitors, such as the PRMT5 selective inhibitor (EPZ015666), have been generated and demonstrate promising therapeutic results against specific cancer types in pre-clinical trials (Chen et al. 2017). Enzymes for histone methylation, demethylation, and readers are summarized in Tables 3 and 4.

**Table 3:** Drugs against histone lysine methylation changes

	Writer (KMTs)	Reader	Eraser (KDMs)
Enzyme	KMT1 (SUV 39H1, SUV 39H2, G9a, GLP, SET DB1, SET DB2) KMT2 (MLL 1–5, hSET1A, hSET1B, ASH2) KMT3 (SET2, NSD1, SMYD1-3) KMT4 (**DOT1L**) KMT6 (EZH1, **EZH2**) KMT7 (SET7/9) KMT8 (PRDM2/RIZ1)	MBT family PHD fingers proteins Chromodomain proteins PWWP domain proteins WD40 repeat proteins	**KDM1** (**KDM1A**, KDM1B) KDM2 (JHDM1A, JHDM1B) KDM3 (JHDM2A, JHDM2B) KDM4 (JMJD2A-2D) KDM5 (JARID1A-1D) KDM6 (UTX, JMJD3) KDM7 (JHDM1D, PHF2, PHF8)
Drugs	EPZ6438 (phase I/II) EPZ5676 (phase I)		ORY-1001 (phase I/IIa) GSK2879552 (phase I)

Enzymes for the drug target are highlighted in bold

**Table 4:** Drugs against histone arginine methylation changes

	Writer (PRMTs)	Reader	Eraser
Enzyme	PRMT1, PRMT2, PRMT3, PRMT4, **PRMT5,** PRMT6, PRMT7, PRMT8, PRMT9	Tudor domain proteins	JmjD6 PAID
Drugs	EPZ015666 (preclinical)		

Enzymes for the drug target are highlighted in bold

## Histone modification-phosphorylation, ubiquitination, and histone variant

Protein phosphorylation is a very important PTM involved in many cellular processes. Proteins with specific amino acid residues, such as serine, threonine, and tyrosine residues, are phosphorylated by a protein kinase by the addition of a covalently bound phosphate group. Phosphorylation alters the structural conformation of a protein, causing the target protein to become either activated or deactivated. Protein kinases and phosphatases work independently and balance modifications to regulate the function of proteins. The most well-characterized histone phosphorylation is H3S10. S28 and T11 phosphorylation are known for transcriptional regulation and mitosis. Aurora B kinase, mitogen and stress-activated protein kinases 1 and 2 (MSK1 and MSK2), Ribosomal s6 kinase 2 (RSK2) IκB kinase-α (IKK-α), or PIM1 kinase can phosphorylate H3S10 in a DNA-context manner upon different stimuli for immediate-early gene expression (Nowak and Corces 2004). In fact, several studies reported that Aurora B is overexpressed in a variety of human cancers, particularly in colorectal and breast cancer (Ota et al. 2002; Tanaka et al. 2008). Histone phosphorylation, in cooperation with other histone modifications, plays a crucial role in DNA damage response pathways and participates in recruitment of downstream DNA damage response and repair proteins, as well as in the amplification of DNA damage signals. The histone H2A variant, H2AX, is rapidly phosphorylated at S139 by ATM, DNA PK kinases, or ATR upon DNA damage stresses and spread over megabases from the break site, which serves as a platform for recruiting other DNA damage response proteins, including 53BP1 (p53-binding protein 1), BRCA1, and NBS1 (Turinetto and Giachino 2015). H2AX gene is frequently lost in cancer, and H2AX deficiency can lead to increased sensitivity to ionizing radiation, which exhibit genomic instability and enhanced susceptibility to cancer (Georgoulis et al. 2017). However, epigenetic drugs targeting histone phosphorylation have yet to be established.

Ubiquitin is a small (8.5 kDa) regulatory protein, and ubiquitination is the addition of ubiquitin to the lysine residue of a substrate protein. The most well-known role of protein ubiquitination is to degrade target protein primarily via the proteasomal degradation pathway (Swatek and Komander 2016). Histones, especially H2A and H2B, are well-known substrates for ubiquitination. All four histones and linker histone H1 can be ubiquitinated, and a single ubiquitin moiety conjugated to H2A-K119 (ubH2A) and H2B k120 (ubH2B) is the most dominant form. There are several histone ubiquitin ligases and deubiquitinating enzymes (DUBs) identified, and histone ubiquitination plays critical roles, including transcription, maintenance of chromatin structure, and DNA repair (Cao and Yan 2012). H2Bub is highly associated with active gene expression, while H2Aub plays a role in transcriptional silencing with other repressive histone modifying enzyme complexes, such as polycomb repressive complex 1 (PRC1) (Minsky et al. 2008; Zhou et al. 2008). Conversely, H2A DUBs are often required for gene activation, indicating the importance of histone ubiquitination in gene expression (Joo et al. 2007; Zhu et al. 2007). Histone ubiquitination also plays an important role in DNA damage. When DNA damage causes DNA double-strand breaks (DSB), histone variant H2AX is rapidly phosphorylated and recruits DNA damage response regulators followed by subsequent recruitment of histone ubiquitin ligases RNF8 and RNF168, which catalyze the K63-linked polyubiquitination chain formation of histone H2A and H2AX (Uckelmann and Sixma 2017). Aberrant histone ubiquitination, such as down-regulated H2Aub and H2Bub, was found in several cancers (Zhu et al. 2007; Prenzel et al. 2011). To date, there are no therapeutic reagents targeting ubiquitination or deubiquitination. Very little is known about histone modification through the small ubiquitin-related modifier (SUMO) or neddylation. SUMO shares 18% identity with ubiquitin, and NEDD8 is 90% homologous to ubiquitin. Histone H4 can be modified by SUMO family proteins and can associate with transcriptional repression through recruitment of HDAC1 and HP1 (Shiio and Eisenman 2003). Histone neddylation to H2A antagonizes H2A ubiquitination, which negatively regulates DNA damage repair pathways (Li et al. 2014). Although SUMO or Nedd8 share a similar structure with ubiquitin, they play distinctive epigenetic roles in cooperation with other modifiers, indicating the complexity of regulating the epigenetic process.

Histone variants are proteins that substitute for the core canonical histones (H3, H4, H2A, H2B) in nucleosomes in eukaryotes and often confer specific structural and functional features. Unlike epigenetic regulation of ‘canonical’ histones through posttranslational modification, histone variants work through specific deposition and removal machineries. They have important roles in early embryonic development, chromosome segregation, transcriptional regulation, DNA repair, and other processes. There are interesting reports for histone variants macroH2A1 association with cancer. Reduction of macroH2A1.1 protein is negatively associated with lung cancer recurrence, and later reports have shown that alternative splicing of macroH2A1 regulates cancer cell proliferation (Sporn et al. 2009; Novikov et al. 2011). There is a growing awareness that histone modifications and chromatin organization influence pre-mRNA splicing and its epigenetic role in cancer (Khan et al. 2012). It suggests that not only epigenetic modifying enzyme, but also the enzyme for pre-mRNA splicing could be epigenetic therapeutic targets.

## Conclusion

**Fig. 1:**
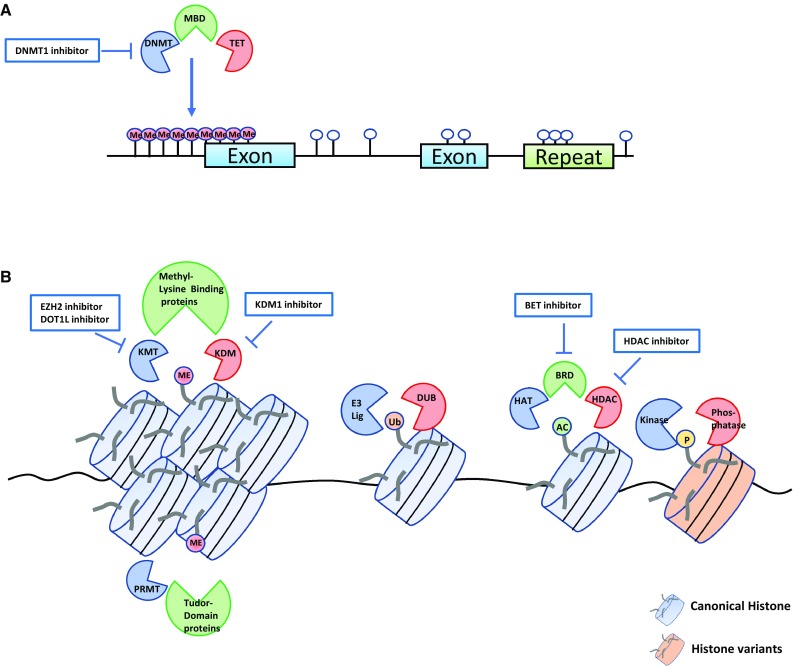
Graphic summary of epigenetic alterations involved in cancer and available drugs targeting epigenetic mechanisms. **a** Tumorigenesis through aberrant methylation of CpG islands. DNA methylation can be written by DNMTs (in blue), recognized by MBD proteins (in green) and erased by TET proteins (in red). Epigenetic drugs targeting DNMT1 are approved by the FDA. **b** Tumorigenesis through aberrant histone modifications. Writers of each histone modification such as histone lysine methyltransferase (KMT), histone acetyltransferase (HAT), ubiquitin E3 ligases (E3 lig), protein arginine methyltransferase (PRMT), kinase are shown in blue. Readers such as methyl-lysine binding protein, tudor domain protein, bromodomain and extra terminal domain family member (BRD) are shown in green. Erasers such as histone deacetylase (HDAC), histone lysine demethylase (KDM), and deubiquitinating enzyme (DUB), phosphatase are shown in red. Canonical histone is shown in blue and histone variants is shown in brown. KMT inhibitors, KDM1 inhibitors, BET inhibitors, HDAC inhibitors are either approved or under clinical trials. Apart from the targets shown here other possible epigenetic targets for drug development are also available. *AC* acetylation, *ME* methylation, *Ub* ubiquitination, *P* phosphorylation

Involvement of epigenetic factors in cancer development is now widely accepted. We have accumulated vast knowledge on how epigenetic aberration can affect cancer initiation, progression, and metastasis. In this review, we have discussed basic epigenetics and its alteration in cancer as well as available drugs targeting epigenetic mechanisms. Major epigenetic modifications as a cancer target are summarized in Fig. 1. We focused on DNA methylation (Fig. 1a) and histone modification (Fig. 1b) among various other mechanisms and summarized current studies regarding how genetic alteration is linked to abnormal epigenetic changes. We should note that each epigenetic modification is not a separate or mutually exclusive event, but rather they are networking with each other to cause subsequent changes. For example, double-strand break from DNA damage rapidly enhances histone H2A and H2AX phosphorylation. In addition, other histone modifications, such as histone acetylation, and ubiquitination follow for further recruitment of DNA damage repair regulatory proteins.

Along with the accumulation of knowledge about the biology and function of epigenetic modifications and their regulatory mechanisms in cancer, four anti-cancer drugs that target these mechanisms have been currently approved, and many others are in clinical trials. However, use of these drugs have a few limitations. As most of the histone modifying enzymes have several different substrates, use of enzyme inhibitors can have limitation in substrate specificity. Conversely, targeting non-histone proteins for cancer therapy can be another strategy for cancer drug development. As cancer results from a series of genetic and epigenetic molecular events, overcoming the disease would need the use of a combination of multiple genetic and epigenetic targets. To date, the only approved epigenetic anticancer agents are HDAC inhibitors and DNMT inhibitors. Our next challenge is to develop additional drugs targeting other classes of epigenetic enzymes and to attempt combinations with those developed to achieve better substrate and cancer specificity.
